# Supplementary material for: Are carbon emissions trading and green financial instruments synergistic? -Comprehensive quantitative research based on content analysis
Source: PLoS One. 2024 Mar 7;19(3):e0298601. doi: 10.1371/journal.pone.0298601 (PMC10919601; doi:10.1371/journal.pone.0298601)
Supplement: S1 Data — (ZIP) [file pone.0298601.s001.zip › Data/地级市Z_F工作报告.zip!/╣π╢1⁄2/╖≡╔╜╩╨/└┤╫╘╣1⁄2╓┌║┼í╛┬φ┐╦╩2╛▌═°í┐_2015.docx]

各位代表：

　　现在，我代表佛山市人民政府向大会报告政府工作，请予审议，并请政协各位委员和其他列席人员提出意见。

2014年工作回顾

　　2014年，是贯彻落实党的十八届三中全会精神，全面深化改革的开局之年。市政府在省委、省政府和市委的正确领导下，在市人大、市政协的监督支持下，积极应对复杂形势，坚定不移落实“稳增长、促改革、调结构、惠民生、防风险”的各项政策措施，求真务实、锐意进取，各项工作取得新进步。

　　——经济实力得到增强。全年实现地区生产总值7603.28亿元，增长8.6%；　人均地区生产总值103825元。全社会固定资产投资2612.45亿元，增长15.0%。社会消费品零售总额2560.58亿元，增长13.1%。进出口总值688.18亿美元，增长7.6%，其中出口467.20亿美元，增长9.9%。

　　——质量效益得到提升。实现财政总收入1579.01亿元，增长6.2%。地方一般公共预算收入500.73亿元，增长14.3%。先进制造业和高技术制造业分别占全市工业总产值比重36.1%、6.9%，同比提高0.3、0.6个百分点。

　　——城市环境得到改善。城市升级三年行动计划基本完成，城市形象品质得到较大提升。全市PM2.5下降15%、PM10下降21%[1]。单位GDP能耗预计下降4.25%，主要污染物二氧化硫和化学需氧量排放量完成省下达的削减目标。

　　——人民生活得到实惠。全市财政民生支出337.39亿元，占地方一般公共预算支出的64.3%。城镇新增就业8.2万人，城镇登记失业率2.35%。城镇、农村常住居民人均可支配收入预计分别增长9.0%、9.6%。城乡居民储蓄存款余额5806.94亿元，比年初增长3.7%。

　　一年来，主要做了以下工作：

　　一、加大产业转型力度，结构调整取得实效

产业转型有新进展。工业结构调整提速。省委、省政府要求佛山作为珠江西岸先进装备制造产业带龙头，我们抢抓机遇，迅速行动，启动万亿规模先进装备制造业产业基地建设。积极落实省技术改造扶持政策，设立市优质技改创新项目贷款风险补偿基金，完成工业技术改造投资278.56亿元，增长23.7%。推进“两化”深度融合[2]，22家企业获批国家、省“两化”融合管理体系贯标[3]试点企业，27家企业入选广东省电子商务百强。一汽大众二期、福田汽车[4]、南车制造基地等项目陆续开工，阿格蕾雅光电材料、亨氏食品、村田纳米陶瓷电容器材等项目投产。现代服务业发展动力增强。出台扶持服务业特别重大项目实施办法，南海万达广场、万科广场开业，中国慧聪家电城及家电博物馆项目运营，季华中央商务带、桂澜路千米商贸长廊建设加快，佛山华强广场动工，国通保税物流中心（B型）获批。广东工业设计城、顺德创意产业园成为全省有影响的工业设计产业集群区域。长鹿旅游休博园获评国家5A级旅游景区，佛山（国际）家居博览城、桂城平洲玉器街、乐从国际会展中心、高明盈香生态园成为国家4A级旅游景区。成功举办第十届中国（佛山）机械装备展览会、第七届中国（顺德）工业设计博览会。农业现代化步伐加快。出台农民专业合作社三年发展计划，新增农民专业合作社44个、市级农业龙头企业20家、现代农业园区18个。

　　招商引资有新成果。引进超千万美元的外资签约项目98个，投资总额82.88亿美元。引进超亿元的内资签约项目147个，投资总额963.23亿元。福田汽车、风发科技智能电机生产基地等项目，本田汽车零部件、萨克米机械、爱信精机等外资龙头企业增资扩产项目，广东集成芯片研发与产业培育中心、广东化工交易中心、浪潮云计算中心等现代服务业项目签约落户。重大平台建设有新进展。佛山国家高新区引进阿里巴巴·佛山产业带、大金智地高端产业服务区等项目，佛山科技街初具雏形。中德工业服务区引进及洽谈德国安联财险、瑞曼迪斯集团、库卡机器人集团等项目。广东金融高新区引进广东金融结算中心等54个项目，投资及募集资金90多亿元。南方智谷、智慧新城、绿岛湖都市产业区、佛山泛家居电商创意园、中欧科技合作产业园、广东新光源产业基地、广东智能制造产业基地、广东物联网应用产业基地、佛山塑料制品国际采购中心等平台建设和招商顺利推进。

　　金融科技产业融合有新亮点。全市财政安排创新型城市建设资金21.07亿元，带动规模以上企业研发投入增长。我市获批国家知识产权示范城市、国家知识产权服务业集聚发展试验区、全国陶瓷产业知名品牌创建示范区，南海区获批全省金融科技产业融合创新综合试验区，顺德区获批国家知识产权试点城市。公共创新平台建设加快，顺德中山大学卡内基梅隆大学国际联合研究院、南海广工大数控装备协同创新研究院启用，三水合肥工业大学研究院挂牌成立，佛山中科院产业技术研究院新材料产业园投入使用。新增国家认定企业技术中心3家、国家级科技企业孵化器4家；　国家高新技术企业73家，总数达618家；中国驰名商标16个，总数达134个；集体商标[5]4件，总数达21件；广东省名牌产品187个，总数达399个；百万人口发明专利申请量995件，增长55.2%。新增省级、市级创新团队13个，省级、市级工程中心96家，引进国家“千人计划”专家23人，设立院士工作室29个。金融服务实体经济能力提升。市、区财政出资5亿元设立支持企业融资专项资金，帮助中小企业缓解融资困难。成立佛山科技金融综合服务中心，设立市科技型中小企业信贷风险补偿基金、产业金融引导基金，启动佛山科技保险试点。成立市金融发展与稳定领导小组，防范和化解企业财务风险，优化金融生态环境。金融创新有新进展。广东金融高新区股权交易中心挂牌企业融资38.6亿元，佛山民间金融街引入60多家金融机构。全市新增银行2家、保险公司4家、小额贷款公司3家、股权投资企业56家、融资租赁公司10家、新三板企业11家、上市企业2家。

　　民营经济做强做大有新成绩。民营工业占全市规模以上工业总产值比重69.6%，比上年提高3.7个百分点，对全市工业增长贡献率79.8%。完善各级领导挂点重点培育企业工作机制，实施“一企一策”帮扶，研究解决重点培育企业发展难题。认真落实中央“营改增”、小微企业减税等政策，全市减免各项税收122.09亿元。鼓励实施“两化”融合、机器人应用、品牌营销等举措，加强重点企业培育，全市产值超千亿元企业1家、超百亿元企业11家。举办民营企业精英研修班，引导中小民企建立现代企业制度，2010户个体工商户转型升级为企业。

二、全面推进城市转型升级，城市形象品质不断提升

城市升级成效突出。“强中心”战略深入实施。禅城老城区传统文化氛围浓厚，“大祖庙商圈”初具规模，岭南天地三期加快建设，梁园周边环境改造提升初步完成，仁寿寺改造顺利推进，汾江河“一河两岸”景观提升工程完成；禅西新城绿岛湖行政服务大厅、欧洲工业园A区启用，人才公寓、国际中学等建成使用；桂城千灯湖片区金融公园、灯湖广场等项目建设顺利；佛山新城中欧（中德）服务中心等平台主体建成，中德高技术实验园研发楼及人才公寓即将竣工，交通枢纽中心完成地下基础工程。城市组团建设成效显著。狮山博爱湖、南海市民服务中心等城市配套项目进展顺利，西樵听音湖片区雏形显现；　顺德新城保利商务综合体、美的广场、置业广场封顶，华侨城文化旅游综合项目加快推进；西江新城基本完成核心区示范工程建设，明湖公园、秀丽河公园、丽江水廊建成；三水新城基础设施建设加快推进。“三旧”改造稳步实施，荣获全省“三旧”改造一等奖，北江新区“三旧”综合改造项目、高明江滩滨河景观工程成为样板。

　　基础设施建设力度加大。广明高速陈村至西樵段、肇花高速三水段正式通车。市域路网中禅西大道二期、G321南海段改造、三花公路改造、南庄至九江公路复线、佛陈大桥扩建和樵乐路、龙湾大桥、季华路快速化改造完工。贵广、南广铁路佛山段通车，三水南站投入使用。佛山西站、佛肇城际线、广佛地铁二期加快建设，城市轨道交通二号线一期、南海新型交通系统试验段工程开工，城市轨道交通三号线工程开展前期工作。能源、水利、通信宽带、智能电网等建设加强。国家重点项目500千伏“西电东送”换顺线工程[6]、500千伏东坡变电站、16个110千伏以上输变电项目投产。光纤入户率达34.5%，新增4G基站8322个。成为首批国家信息惠民试点城市。

　　城市管理机制不断完善。严格落实城市管理考核机制。开展城市道路环境卫生、建设工地和泥头车污染源头管控、户外广告清理、水浸黑点治理等专项整治。整合数字城管资源，建立渣土运输GPS监控平台和工地视频监控平台。推动城市管理向乡村延伸，实施农村整治“1060”工程[7]，切实改善乡村面貌和环境卫生状况。提升城市公共交通服务水平，禅南、禅顺公交一体化提速。全市新增49条公交线路、993辆公交车、4800辆公共自行车、54个公共自行车服务站点，建成38个公交首末站和枢纽站。中心城区公交分担率提升至30.1%。

　　环境治理和生态建设成绩显著。建立环保责任考核追究机制，设立“环保警察”，强化环保执法。基本完成100项环保民生实事。全力推进大气污染防治。扩大限燃区域至中心城区面积60%；深化工业企业大气污染治理，整治污染企业804家；严厉打击黑烟车，全年淘汰黄标车及老旧车8.05万辆；严格控制扬尘污染，征收扬尘排污费。坚持“一河一策”治理水环境污染，19条重点整治河涌基本达到V类标准。新建污水配套管网217.84公里。对禁养区内畜禽养殖场全面清理。强化固体废弃物、危险废物管理，积极推进南海垃圾焚烧发电一厂等改扩建，大力开展城镇生活垃圾无害化处理。新增国家级生态乡镇7个，总数达19个。新建和改造提升绿地面积720.73公顷，创建绿色建筑175万平方米。建成智慧公园、九江外滩景观工程、丽江水廊等10个大型公园，建设和改造提升一批村居公园和社区体育公园。完成珠二环高速顺德段、东平河两岸等106公里生态景观林带建设。谢边互通立交、顺峰山东入口等重要节点景观改造基本完工，完成33.9公里“五位一体”[8]沿街景观综合整治。

　　三、深入推进改革开放，城市发展活力增强

　　市场活力进一步激发。编制实施企业投资准入负面清单、审批清单和市场监管清单。建立以备案制为主的企业投资管理体制。商事制度改革成效显著，全市新登记市场主体7.29万户，工商登记“全城通办”进展顺利。在省内率先推出政府权责清单。网上办事大厅建设提速，市、区两级网上服务事项4971项，二级以上办事深度事项占97.43%，三级占56.21%[9]。设立616台“市民之窗”自助服务终端，39万笔行政服务通过终端办理。公共资源交易一体化服务平台建设顺利，市公共信用信息管理系统初步建成。成为省建设法治化国际化营商环境试点城市。

　　基层社会治理创新稳步实施。开展“城乡社区建设提升年”活动，社区协同共治、社区服务水平不断提升。禅城区开展社区“一门式”服务试点，南海区开展社区网格化管理试点，三水区推行“一站式”公共服务平台。顺德区试点建立社会组织综合监管体系。各区均成立社会组织孵化培育基地，全市社会组织达4663家。成立社工人才培育基地、市社工协会和市志愿者联合会。全市村（居）完成换届选举工作，书记、主任“一肩挑”比例达89.9%。

　　农村综合改革扎实推进。全面完成农村集体经济组织证书颁发和农村集体土地所有权确权登记发证。禅城区完成农村“股权固化”，南海区稳妥推进确权确股式股权改革，三水区和顺德区均安镇、容桂街道试点开展农村土地承包经营权确权登记颁证工作。全市农村财务网上监控平台和集体资产交易平台交易资产累计7.7万宗，合同标的总额426.09亿元。高明区推进“一事一议”财政奖补计划[10]。三水区探索实施村民自治决策、执行、监督分开的“两级议事会”治理模式。

　　开放合作水平不断提升。全市境外投资项目26个，投资总额增长283.2%。实际利用外资26.56亿美元，增长5.4%。顺德区获批国家级出口家电质量安全示范区。广佛同城化取得新进展，交通路网和轨道交通对接进展顺利，两市金融机构资金结算技术上实现同城化，环保协作、民生合作不断深化。佛港澳合作有新成效，汇丰环球运营中心、广发金融中心一期投入运营。荣获国际友好城市交流合作奖，与德国因戈尔施塔特市、波兰斯达洛加勒德市结为国际友好城市。建立对口帮扶云浮工作新机制，举办7场招商会，投入对口帮扶云浮财政资金4.1亿元，加快佛山（云浮）产业转移工业园建设。扶贫开发“双到”完成年度任务。对口援建新疆伽师、西藏墨脱取得新成效。

　　四、大力发展民生社会事业，促进社会和谐进步

民生保障有新提高。省、市民生实事全面完成[11]。企业退休职工月人均基本养老金增长14.2%，城乡居民社会养老保险基础养老金增长12.5%。城乡低保标准提高8.5%，城乡低保对象大病医疗救助报销比例平均达80%，农村五保供养标准平均增长26.4%。教育综合改革试验区建设加快，成为全省首个推进教育现代化先进市，全市五区在全省率先通过全国义务教育发展基本均衡区国家督导评估。实施技能晋升培训补贴，5.1万人参加技能培训。实施创业带动就业政策，发放创业小额担保贷款5616万元，成功创业6662人，带动就业3.6万多人。“零就业”家庭持续动态“清零”。加快构建“基层首诊、分级诊疗、双向转诊”模式，建立疾病应急救助制度，鼓励和引导社会办医，新增非公立医疗机构57家。新市民入户及随迁子女就读义务教育公办学校实施统一积分，符合条件异地务工人员随迁子女参加居民医保享受同等财政补贴，实行异地务工人员大病救助。

　　文化活动魅力彰显。重点公共文化设施建设加快，市图书馆新馆投入使用，市文化馆新馆封顶，佛山大剧院加快建设。祖庙、仁寿寺、梁园等改造提升工程加快推进。成功举办中国四大名镇[12]城市交流活动、佛山秋色欢乐节和亚太地区龙舟公开赛等活动。“魅力佛山·四季情韵”艺术惠民工程精彩纷呈。顺德区被联合国教科文组织评为“世界美食之都”。对外文化交流广泛开展，龙狮表演、剪纸、陶艺、香云纱等佛山非物质文化遗产在德国、澳大利亚等国展示。加强社会主义核心价值观宣传教育，开展“微文明”、“微志愿”活动，创建全国文明城市深入推进，市民文明程度不断提升。

　　平安佛山建设有新举措。基层“平安村居”和各行业“平安细胞”创建覆盖面80%。公安“六大专项”打击整治行动获得全省第2名，110刑事警情同比下降10%。禅桂新中心城区“禁摩禁电” [13]平稳推进。出租屋管理进一步强化和规范。开展“全国安全社区”创建和基层安全生产网格化监管试点，里水、大沥、北滘、伦教镇被评为“全国安全社区”，新增安全生产达标企业6272家，总数达19251家。食品（农产品）安全示范市建设扎实推进。试点推行家禽“集中屠宰，生鲜上市”，打造肉食品“全链条闭环式”监管模式，新增餐饮服务食品安全示范点100家、阳光厨房695家、市级“菜篮子”基地19个、省级食品安全示范学校食堂100家。强化疫情联防联控，有效遏制登革热、H7N9禽流感、手足口病等疫情。

　　五、全面启动人民满意政府建设，政府自身建设得到加强

“六个政府”[14]建设扎实推进。出台《佛山市建设人民满意政府行动方案（2014-2020年）》和11个行动子计划，筹措项目资金76.1亿元，其中197项民生项目完成145项。建立人民满意政府第三方评价体系。根据第三方调查评估，2014年佛山市建设人民满意政府总体评价82.46分。

　　依法行政不断深化。以市重大行政决策程序规定为基础，完善重大行政决策配套制度和程序。健全市政府法律顾问制度。严格执行市人大及其常委会的各项决议决定，自觉接受市人大及其常委会的法律监督和市政协的民主监督，认真办理人大代表和政协委员的建议、提案273件，办复率100%。创新行政复议机制，全面推广集中复议制度。根据中国政法大学发布的中国法治政府评估报告，佛山法治政府建设排名第3。

　　深入整治“四风”问题。认真开展党的群众路线教育实践活动，切实改进作风，全市会议数量下降2.4%、文件数量下降6.1%。深入推进市级部门预算、决算和“三公”经费预算、决算公开，市级财政预算资金全部纳入国库集中支付。建立镇（街）领导干部每周定期直接联系群众制度。构建全省首个狠刹“四风”网络监督平台，打造民主评议政风行风、领导干部操办婚丧喜庆事宜信息管理等平台，出台领导干部问责实施意见和行政过错责任追究实施细则，强化对领导干部管理和监督。

　　老年人、残疾人、妇女儿童事业，审计、粮食储备、民族宗教、双拥优抚、台湾事务、档案方志、社会科学、人防、气象等工作取得新成绩。

　　各位代表！过去的一年，成绩来之不易。在此，我代表佛山市人民政府向全市广大干部群众，异地务工人员，中央、省驻佛山单位，驻佛山人民解放军指战员，武警官兵和社会各界人士表示崇高的敬意！向长期关心支持佛山建设的港澳台同胞、海外侨胞、国内外友人表示衷心的感谢！

　　我们清醒地认识到，我市经济社会发展仍然存在一些困难和问题，主要是：经济运行不稳定因素较多，稳增长任务艰巨；产业转型升级任重道远，现代服务业发展有待提升；创新驱动发展的基础不牢固，自主创新能力整体不强；企业不同程度存在经营困难；城市优质公共服务供给有待改进；环境治理任务较重，大气和水污染问题较为突出；“12·31”事故教训极其深刻，安全生产企业主体责任和政府监管责任亟需强化。对此，我们将在今后工作中深入研究，认真解决。

2015年工作安排

2015年是全面深化改革的关键之年，是全面落实依法治国方略的开局之年，也是全面完成“十二五”规划的收官之年。我市发展面临许多有利因素，领衔打造珠江西岸先进装备制造产业带，第三产业发展成效逐步显现，一批重点项目和高端平台建设加快，经济发展后劲增强。城市形象品质不断提升，各项改革不断深入，综合环境持续优化。总体而言，我市发展处于重要战略机遇期，我们要认清形势、坚定信心，适应新常态、把握新机遇，坚定不移促进经济发展方式转向质量效率型集约增长，经济结构转向存量优化、增量优质的深度调整，对全省经济增长和结构调整起支撑作用，争当全省改革发展排头兵。

　　2015年政府工作的指导思想：深入贯彻落实党的十八大，十八届三中、四中全会，中央经济工作会议和省委十一届四次全会精神，按照市委十一届六次全会的总体部署，主动适应经济发展新常态，坚持稳中求进工作总基调，以经济提质增效升级为中心，以创新驱动为总抓手，以建设人民满意政府为动力，保持经济运行在合理区间，推进依法行政，提升开放水平，狠抓改革攻坚，加强民生保障，强化风险防控，完成“十二五”发展目标，规划“十三五”发展任务，努力推动经济平稳健康发展和社会和谐稳定。

　　2015年经济社会发展主要预期：全市地区生产总值增长8.5%左右，人均地区生产总值增长7.8%；地方一般公共预算收入增长10%；全社会固定资产投资增长15%；社会消费品零售总额增长12%；进出口总值增长3.8%；　城乡居民收入增长与经济增长基本同步；城镇登记失业率控制在3%以内。

　　实现上述目标任务，重点做好以下八方面工作：

　　一、抢抓打造珠江西岸先进装备制造产业带机遇，加快产业转型升级

积极研究“工业4.0” [15]计划，结合佛山实际，推动佛山制造智能化转型。坚持先进装备制造业和现代服务业双轮驱动，力争经济结构战略性调整走在全省前列。

　　大力发展先进装备制造业。积极落实省政府珠江西岸先进装备制造产业带布局规划，集聚发展智能制造、节能环保、新能源、汽车制造和装备服务业等重点装备产业，力争到2020年装备制造业总产值突破1万亿元，努力建设世界级先进装备制造业产业基地。着力引进一批带动能力强的龙头项目，培育一批拥有核心部件制造技术的“高、精、专”中小型配套企业，促进装备制造业向数字化制造、绿色制造和智能制造跨越发展，加快构建装备制造业全产业链。全力推进落实重点支撑项目[16]和珠海签约10个项目[17]，加快一汽大众二期、福田汽车、南车制造基地、陆地方舟新能源汽车等项目建设，推动项目落地，形成产能。

　　力促现代服务业发展提质增效。全面落实提升发展第三产业各项政策措施，重点支持发展金融、现代物流、商务会展、工业设计、服务外包等生产性服务业，加快发展医疗健康、教育培训、养老服务、文体休闲等生活性服务业。加快推进国药集团中药南方总部、苏宁电子商务运营基地、华侨城文化旅游综合项目等145个重大服务业项目，14个省级服务业集聚区建设，规划建设一批总部经济基地，促进第三产业发展提速、比重提高、水平提升。大力发展文化艺术、创意设计、动漫游戏等文化产业，加快建设石湾“国家级文化产业示范园区”等重点项目，促进社会资本、金融资本和文化资源融合，将文化旅游创意产业打造成为我市战略性支柱产业。

　　以技术改造为核心促进存量优化。贯彻落实省、市扶持技术改造优惠政策，积极争取省财政技术改造专项资金补助，市、区财政3年配套24亿元支持企业技术改造。实施全市工业企业技术改造行动计划，未来3年全市工业技术改造投资计划完成1300亿元，年均增长25%，力争半数规模以上工业企业完成新一轮技术改造。整合运用物联网、移动互联网、云计算、大数据、3D打印技术等新一代信息技术和高端智能制造技术，加快机器人推广应用，实施“百企智能制造提升工程”、“自动化生产线改造”计划，推动佛山制造业向智能化转型，力争成为全省乃至全国智能制造先进城市。实施“两化”深度融合战略，加强云制造公共服务平台推广运用，促进更多制造企业“登云上线”。推动企业采用卓越绩效管理、精益生产管理等先进质量管理方法，制定产业联盟标准，培育细分行业龙头，打造行业区域品牌，提升产品质量和企业效益。

　　以产业链招商为抓手促进增量优质。树立“大招商”、“招大商”、“择优择强”的招商理念，进行高水平、专业化的招商策划，把先进装备制造业、战略性新兴产业和现代服务业作为招商重点，瞄准国内外先进地区和城市实施定向招商和主题招商，着力引进一批高端优质项目、先进技术、关键设备和优秀人才。充分挖掘本土企业增资扩股潜能，重点推动我市龙头企业与国际装备业巨头开展合资合作，带动引进一批机器人系统集成商，建设机器人产业园。不断提升佛山国家高新区、中德工业服务区、广东金融高新区等重大平台的产业内涵和建设水平，加快南方智谷、智慧新城、绿岛湖都市产业区、广东新光源产业基地、广东智能制造产业基地、珠江西岸装备制造产业创新基地、中科院佛山新材料产业基地等产业平台建设，增强承接吸纳国内外优质项目和高级人才的能力。

　　做强做优民营经济。落实推动民营企业跨越发展各项政策，发挥民营企业在打造先进装备制造产业带中的主力军作用，发展壮大产值超百亿元企业队伍。坚持各级领导挂点企业制度，定期深入企业，帮助企业破解发展难题。完善中小微企业政策服务体系，鼓励和引导中小民营企业进行公司制、股份制改造。推动民营资本与国际资本、战略性新兴产业对接，形成新的竞争优势。继续开展高级经理人、企业接班人培训。

　　二、大力实施创新驱动发展战略，促进金融科技产业深度融合

把创新驱动发展战略作为经济工作的总抓手，努力建设自主创新示范区，当好全省创新驱动发展排头兵。

　　提升科技创新能力。以创建国家创新型城市为引领，大力实施工业创新驱动转型升级攻坚战三年行动计划。强化企业自主创新主体作用，实施大中型骨干企业研发机构全覆盖，支持美的等大型企业与科研院所联合组建研究院，各区重点帮扶一批创新型、科技型骨干企业。深化产学研协同创新，加快建设中科院产业技术研究院、华南智能机器人创新研究院、华南家电和机械检测基地、广东半导体照明产业联合创新中心、诺尔贝机器人研究院等重大新型研发机构。加快佛山知识产权交易平台建设，积极申报中国佛山(陶瓷）、顺德（家电）知识产权快速维权中心，全面推进国家知识产权示范城市和国家知识产权服务业集聚发展试验区建设。

　　加强金融创新发展。充分发挥金融支持实体经济发展的作用，优化金融生态环境，引导民间资本投入科技型、智慧型企业。加快建设省金融科技产业创新融合试验区和产业金融改革试验区，完善“政策金融、科技金融、民间金融”三位一体金融服务模式，充分发挥佛山民间金融街、广东金融高新区股权交易中心、深交所路演中心等平台作用，创新发展互联网金融、知识产权证券化、众筹融资、融资租赁等金融产品和服务，构建科技金融服务体系。

　　实施人才强市战略。完善人才政策，重点引进和培养创新科研团队、创新领军人才、高技能人才和企业经营管理人才等高层次人才。加快“产业社区”建设，完善产业园区基础设施和生活服务配套，努力解决人才居住、子女入学等问题。实行招商引资和招才引智相结合，在引进中高端企业项目的同时，注重引进拥有先进技术的人才团队，实现产城人三者良性互动。

　　三、加快新型城镇化建设，推动城市升级向城市升值转变

　　把握建设珠三角世界级城市群机遇，全面提升佛山城市价值和竞争力。

　　实施城市升级两年延伸计划。深化“强中心”战略，打造佛山城市中轴线[18]，加快佛山新城建设、南海千灯湖片区拓展、禅城绿岛湖都市产业区建设和禅城老城区提升。推动城市组团提升发展质量。加快顺德新城、沥桂新城、西江新城、三水新城等片区建设。围绕绿岛湖、博爱湖、桂畔海、明湖、云东海等水道和生态片区，打造一批生态景观带和滨水区。推动城市升级向乡村和社区延伸。实施百村升级计划，推进30个特色古村落活化[19]、30个城中村（旧社区）改造和48个新农村建设，打造一批美丽乡村、宜居社区。加强特色古村落历史风貌及古建筑保护，实现历史文脉延续。

　　加强基础设施建设。加快推进广佛环线（佛山西站-广州南站）、佛肇城际线建设，配合广州地铁7号线延伸线、广佛江珠线前期工作，尽快启动广佛环线（佛山西站-白云机场）、肇顺南城际等项目。推动轨道二号线一期工程、南海新型交通系统建设，启动轨道三号线建设准备。加快建设佛山西站、佛山新城交通枢纽中心等项目。推进高速路网中广明高速广州段、广中江高速顺德段、佛清从高速南段等建设，加快市域路网中魁奇路东延线二期、岭南大道南延线、汾江路南延线、海五路西延线等建设。积极开展佛山新机场建设可行性研究。加强能源、水利、通信、电网建设。推进220千伏顺德奎福输变电站等项目建设，推动光纤入户工程，加快新能源汽车充电桩建设。

　　加大“三旧”改造力度。完善我市“三旧”改造政策，充分调动社会资本参与改造的积极性。支持南海区开展广东新一轮深化“三旧”改造综合试点。提升改造村级工业园区。推进高速公路沿线改造，专项整治高速公路沿线300米范围内旧厂房，再造百里绿色长廊。推进沿街、沿河、沿湖景观“五位一体”改造，开展内街小巷环境综合整治。

　　提升城市综合管理水平。实施城市管理“四化”[20]工程，完善城市管理考核机制，加快数字城管建设。加强道路卫生、绿化管养、污染源头管控、户外广告清理等工作，进一步提升城市整洁度、美观度。推动社会化市政管理模式向城中村、城乡结合区域、主干道两旁延伸。实施大数据战略和智能交通、智慧社区、智慧医疗等项目，加快国家信息惠民试点城市建设。

　　提高城市和市民文明素质。以创建全国文明城市为抓手，推进文明城市“八大环境”[21]建设，强化巡查督办和考评问责机制。培育和践行社会主义核心价值观，引领社会文明新风尚。持续开展“微文明”市民行动，打造佛山“微文明”品牌。支持发展志愿服务，推广南海区桂城街道“社工+志愿者”社区志愿服务模式，积极发动市民参与城市治理。发展公益慈善事业，弘扬乐善好施、扶弱济贫的传统美德。

　　四、加强生态文明建设，共建美丽佛山

对生态环境倍加重视，对资源能源精打细算，走绿色、低碳、可持续的发展道路。

　　打好环境治理攻坚战。以蓝天、净水、绿地成为佛山新常态为目标，落实新《环境保护法》，建立健全环保综合执法体系和跨区域联动协调机制，强化市、区、镇（街）、村（居）四级环保责任，实现源头严防、过程严管、后果严惩。实施内河涌“一河一策”整治计划，重点推进汾江河、西南涌、芦苞涌等广佛跨界河涌综合整治，确保完成整治任务。加大城镇污水管网建设力度，有效提高污水收集率。整治改造小锅炉、VOCs[22]排放企业，实施电力行业超洁净排放，加速淘汰黄标车，依法整治黑烟车，努力实现大气质量状况持续改善。加强农业面源污染控制和养殖场污染治理。加快全市危险废物处理处置能力建设。开展全市供水及水源地优化整合。加快垃圾处理设施建设，改进垃圾分类收运体系，逐步实现生活垃圾减量化目标。

　　大力发展循环经济。推进排污权有偿使用和交易试点，落实电机能效提升等扶持政策，引导企业自觉减排。对重点耗能企业和公共机构开展节能监察，加大节能执法力度。加快低碳能源管网规划建设，扩大低碳能源使用范围。抓好全国电力需求侧综合管理试点城市建设，加大“3C”绿色电网[23]建设力度，推广分布式太阳能光伏发电应用工程，探索光伏发电项目并网规范管理。推进绿色生态示范城区建设，加快新能源汽车推广应用。

　　加强绿色生态建设。发展生态集约、观光休闲农业，加快现代农业园区和海峡两岸农业合作试验区建设，鼓励支持农业龙头企业和农民专业合作社发展，提高农业现代化水平。创建国家森林城市。开展新一轮绿化佛山大行动，实施森林质量提升、城市绿化增量提质、森林公园和湿地公园扩面、绿色生态廊道建设、乡村绿化美化工程，加速构建森林生态屏障、城乡绿地、水系湿地生态、绿色生态廊道体系，确保完成3万亩森林碳汇工程造林任务，河涌绿化整治659公里。加大社区公园、郊野公园、湿地公园、市政公园规划建设力度。

　　五、加强区域合作交流，扩大对外开放水平

以更开放的胸怀加强国内外交流合作，以更开放的举措谋转型、促发展，以更开放的视野推动更高水平的对外开放。

　　增创对外开放新优势。抢抓“一带一路”[24]建设机遇，以经贸往来、科技合作以及文化交流为主要内容，坚持“引进来”和“走出去”并重，积极开拓新兴市场，把佛山打造成为“一带一路”的重要节点。加强与欧美、东盟、非洲、中东、南美等地区在投资贸易、资源开发、工程承包等方面的深度合作，建设高水平的开放型经济。承办好中国（广东）-印尼经贸合作交流会。支持鼓励民间文化交流，采取多种形式开展城市形象推广，提升佛山在海内外的知名度和影响力。积极扩展与海外华人华侨的联系交往，发展与我市产业、文化相匹配的国际友好城市。

　　密切佛港澳台合作。深入落实CEPA和粤港、粤澳合作框架协议，加强与香港、澳门、台湾地区在贸易投资、先进服务业、科技文化、人才教育等领域的合作交流。推动佛港澳服务贸易自由化，大力引进港澳台现代服务业项目，谋划打造粤港合作高端服务示范区[25]，鼓励开展与香港跨境人民币贸易融资业务。加强佛港跨境电子商务合作，加快国通保税物流中心建设。

　　推进区域经济一体化。积极推动广佛同城化，抓好地铁线网规划对接、海华大桥建设、珠江大桥放射线接广佛新干线等项目，加强广佛交界区域基础设施、产业协作、环境治理、城市管理等对接。支持顺德区与南沙加强合作，融合对接广东自贸区。参与泛珠合作、珠江-西江经济带建设，加强与珠江西岸城市交流合作，着力打造粤桂黔高铁经济带合作试验区。加快佛山云浮一体化帮扶，3年投入财政帮扶资金13.5亿元，加大超亿元优质项目招商力度，助推云浮跨越发展，力争对口帮扶工作在全省领先。做好新疆伽师、西藏墨脱对口援建工作。加强全市统筹规划，落实《佛山市推进区域合作工作方案》。

　　六、深化各项改革，充分激发佛山发展活力

全面深化重点领域和关键环节改革，把改革红利不断转化为发展新动能，营造市场化法治化国际化发展环境。

　　加大市场化改革力度。健全企业投资全链条、全流程、全行业的“三单”管理[26]模式。放宽市场准入，鼓励社会资本参与重大基础设施和公共服务领域投资。深化商事制度改革，加强社会信用体系建设，完善配套监管制度，实现企业准入“宽进严管”。推进公共资源交易监管机制改革。规范政府性债务管理，做好国资优债工作。强化财政绩效管理，推进国库集中支付电子化改革，加快建设现代财政制度。

　　加快政府职能转变。推广行政职权标准化建设，实现量权限权。总结推广禅城区“一门式”政务服务改革试点经验，方便市民办事。加强电子政务规划建设，完善网上办事大厅、改革发展市民建言献策平台和12345统一服务平台，加快建设企业专属和市民个人网页。推进工商、质监管理体制调整，深化事业单位分类改革。

　　创新基层社会治理。推进社区公共服务综合信息平台建设，增强城乡社区服务功能，优化社区工作机制，构建社区自治体系。发挥各级社会组织孵化培育基地作用，培育发展和规范管理社会组织，推动社会组织承接政府职能转移和购买服务。健全社工人才评价机制，加强社工专业人才培育基地建设管理。完善有效预防和化解社会矛盾机制，改革行政复议体制和信访工作机制。

　　探索农村综合改革。开展农村土地承包经营权确权登记颁证试点，完善土地征收和补偿安置制度，稳步推进农村土地承包经营权流转，保障农民合法权益。加快推进区、镇（街）、村（居）三级互联互通的农村集体“三资”管理[27]服务平台体系建设。

　　七、加强社会事业发展，切实保障和改善民生

深入实施民生政府建设系列行动计划，确保完成民生政府建设重点项目任务，稳步提高市民生活水平和幸福指数。

　　实施基本公共服务均等化。推进基层基本公共服务均等化实施方案。深化创建国家教育综合改革试验区。启动学前教育第二期三年行动计划，统筹城乡义务教育资源均衡配置，推进普通高中优质多样特色化发展，加快佛山科技学院新校区建设，完善现代职业教育体系，提升特殊教育发展水平，推动民办教育加快发展。提升就业和社会保障水平。落实促进就业政策措施，指导和帮助高校毕业生、就业困难人员等群体就业。加大创业扶持力度，促进大众创业、万众创新。实施以企业为主体的技能提升培训，增加高技能人才总量。完善全征地农村居民与城镇职工养老保险制度衔接机制，建立健全城乡居民基本养老保险待遇调整机制。扩大社保覆盖面，推动社保业务网络延伸到村（居）。推进养老服务体系建设，完善社会福利和社会救助体系。深化医药卫生体制改革，促进优质医疗资源下沉，提高基层医疗卫生服务能力，推行家庭医生式服务。做好迎接国家卫生城市复审工作。加强新市民积分制服务管理，促进异地务工人员市民化。

　　增强城市文化软实力。启动实施文化升级两年行动计划，建设岭南风韵突出、城市形象鲜明的文化导向型城市[28]。健全现代公共文化服务体系，打造“城乡十分钟文化圈”。实施艺术惠民工程，开展“佛山韵律”系列文化活动。承办第九届广东国际旅游文化节，推动佛山历史文化街区申报国家5A级旅游景区，创建城市中央休闲区[29]。加强传统文化保护与利用，促进非物质文化遗产传承与发展。完善体育设施，推进社区体育公园建设，广泛开展全民健身运动，增强市民体质。

　　着力办好一批民生实事。加强保障性住房建设，确保按时完成省下达的住房保障目标任务。投入6.82亿元推进河涌水环境治理等民生水务工程。新建300公里污水处理厂配套管网。实施100项环保民生实事。提高城乡居民基本养老保险基础养老金水平。提高公益性普惠性幼儿园生均公用经费标准和免费义务教育财政拨款标准。建设区、镇（街）、村（居）三级公共法律服务平台，落实一镇（街）一律师顾问团和一村（居）一专职调解员。新增市级“菜篮子”基地10个。加快推进主要公共场所无线局域网（WLAN）惠民建设。加快“市民之窗”自助服务终端布点，实现村（居）全覆盖，推动全天候自助服务区建设。落实公交提升计划，中心城区公交分担率达32%。完成迳口华侨农场2629户危房改造任务。

　　确保社会大局稳定。深入推进平安佛山建设，完善立体化社会治安防控体系，提升市民安全感。做好国防动员、双拥、优抚安置工作，巩固军政军民团结。加强安全生产监管，落实安全生产“一岗双责”和企业主体责任，强化企业安全生产标准化和基层“网格化”安全监管体系，提升社会火灾防控能力和公共消防安全管理水平，遏制重特大安全生产事故。做好食品（农产品）安全示范市创建深化及验收工作，健全“从农田到餐桌”食品安全电子追溯体系，完善食品监管体系、责任体系和检验检测体系，加快食品安全“社会共治”进程。设立“食品药品警察”，加大执法力度。抓好手足口病、登革热、禽流感、埃博拉出血热等传染性疾病防控，防止出现重大公共卫生事件。

　　八、扎实推进人民满意政府建设

巩固群众路线教育实践活动成果，按照《佛山市建设人民满意政府行动方案（2014-2020年）》和11个行动子计划，着力建设为民务实清廉政府。

　　坚持依法行政。牢固树立“治以法尊”理念，自觉维护宪法与法律权威，严格依照法定权限和程序行使权力、履行职责，建设权责明确、便民高效的法治政府。完善重大行政决策法治化程序，制订行政决策目录管理办法等制度规范。加强政府法律顾问工作。推进政务公开。严格规范公正文明执法，健全行政执法和刑事司法衔接机制，推进重点领域综合执法，推动执法重心向基层下移。完善覆盖城乡的公共法律服务网络，大力创建法治镇（街），提升基层社会治理法治化水平。配合做好行使地方立法权准备工作。

　　建设责任政府。完善行政职权清单管理制度，落实政府部门责任制和岗位责任制。健全行政问责制度，完善行政过错责任追究机制，增强行政责任意识，依法正确履行职责。抓好审计监督，推动公共资金、国有资产、国有资源、领导干部经济责任履行情况审计全覆盖。自觉接受人大、政协、社会团体、市民和新闻媒体的监督。

　　加强作风建设。落实中央“八项规定”，切实抓好群众路线教育实践活动整改。落实廉政建设责任制，加大对违法行政、懒政怠政的行政监督、监察力度，强化重点部门和岗位廉政风险防控，推进政府机关廉政风险科技防控平台建设。严控“三公”经费支出和楼堂馆所建设。完善直接联系和服务群众机制，努力实现服务企业稳增长、服务社区促和谐、服务群众保民生。强化政府绩效管理，狠抓各项工作落实。

　　各位代表，把握新常态、激发新动力。展望未来，我们对佛山发展充满信心。让我们在党的十八大和十八届三中、四中全会精神指引下，按照省委、省政府和市委的决策部署，振奋精神，勇于担当，创新有为，为“加快转型升级，建设幸福佛山”而努力奋斗！
